# Ecological and evolutionary trends of body size in *Pristimantis* frogs, the world's most diverse vertebrate genus

**DOI:** 10.1038/s41598-022-22181-5

**Published:** 2022-10-27

**Authors:** Aldemar A. Acevedo, R. Eduardo Palma, Miguel Ángel Olalla-Tárraga

**Affiliations:** 1 Laboratory of Evolutionary Biology, Department of Ecology, Faculty of Biological Sciences, Pontificia Universidad Católica de Chile, Santiago, Chile; 2 Laboratory of Genetics and Evolution, Department of Ecological Sciences, Faculty of Sciences, Universidad de Chile, Santiago, Chile; 3 Grupo de Investigación en Ecología y Biogeografía, Universidad de Pamplona, Pamplona, Colombia; 4 Department of Biology and Geology, Physics & Inorganic Chemistry, Universidad Rey Juan Carlos, Móstoles, Spain

**Keywords:** Ecology, Evolution, Zoology

## Abstract

Body size is a key organismal trait. However, the environmental and evolutionary factors that drive body size patterns at the interspecific level remain unclear. Here, we explored these relationships between phenotype-environment using neotropical frogs of *Pristimantis*, the world’s most diverse vertebrate genus. We analyzed: (a) whether this group follows the Rensch’s rule, a trend of sexual size dimorphism (SSD) to increase with size when males are the larger sex; (b) whether environmental constraints have influenced body size variation; and (c) how the rates of body size evolution have varied over time. Analyses were based on two information sources, the first one including body sizes of ~ 85% (495 species) of known species in the genus, and a second one incorporating molecular phylogenetic information for 257 species. Our results showed that all *Pristimantis* species exhibited marked SSD but did not follow Rensch’s rule. We found that the models that best explained body size in males, females, and SSD contained environmental variations in temperature, precipitation, and elevation as predictors. In turn, body size has evolved toward an optimum, with a decelerating rate of evolution differentiated between the large *Pristimantis* clades.

## Introduction

Body size is perhaps the most visible trait for most organisms. In some cases, body size correlates with different ecological and evolutionary factors^1–5^, which has generated a commonly repeated question: what are the ecological and evolutionary processes that drive body size limits^6,7^. This question has been approached from a few different perspectives, including studies on allometry and trends correlated with body size^8^, relationships between physiological processes and body size^9,10^, and the processes responsible for evolutionary trends in body size^11,12^. Similarly, questions related to the interspecific differences in body size, particularly related to sexual size dimorphism (SSD; i.e., the differences in size between the sexes), continue to be an essential study subject for understanding how the sexes reach different optimal sizes and which ecological and evolutionary mechanisms drive these differences^13^. Sexual size dimorphism is widespread in different animal groups and is variable even between closely related groups^14,15^. SSD varies in terms of its direction due to differences in the body size of different taxa of vertebrate and invertebrates, with SSD patterns tending to lean towards increased body size in males (e.g., some birds and mammals), but also producing inverse cases where the SSD is female-biased^16,17^. This pattern, represented by the increase in SSD with increasing body size in males, is known as Rensch’s rule^18,19^.

The drivers behind the variation in body size and its disparity between males and females have been associated with ecological, life history, and evolutionary factors^20,21^. Therefore, the integration of ecological information (e.g., elevation, temperature, and precipitation) and evolutionary information (e.g., ancestral states and rates of trait evolution) can play a decisive role in understanding the forces driving the variations and evolution of body size between sexes^22,23^. For example, Rensch’s rule assumes that a larger body size in males is associated with a faster evolutionary rate (speed of genetic or morphological divergence in a lineage per million years), whereas a larger body size in females is associated with a slower evolutionary rate^14,15^, with a macroevolutionary trend where male-biased SSD drives the direction of SSD between related species^24^. However, other studies have rejected Rensch’s rule, for example, in studies that have indicated inverse patterns where SSD is female-biased (e.g.,^25–27^. Evolutionary contexts with an isometry between sexes indicate that changes in body size occurred at the same rate and thus equally influenced the evolution of SSD (e.g.,^26,28^, whereas evolutionary contexts with mixed SSD patterns resulted in phylogenetic lineages with species associated with a female-biased SSD and others with a male-biased SSD^24,29^.

Hypotheses associated with classical ecogeographic rules that relate geographical patterns in the variation of biological traits and environmental gradients are important for understanding interspecific patterns of body size-associated traits at different scales and ecological and evolutionary contexts^30^. Climatic variables have been found to be a major driver of broad-scale interspecific body size patterns for different taxonomic groups, including cold-blooded animals e.g.,^31–34^. In fact, two hypotheses have been recurrent related to body size in amphibians, and both have to do with the importance of surface-to-volume ratios on the homeostatic capacity of organisms through its effects on thermoregulation and hydroregulation^31^. According to the “water availability hypothesis”, larger organisms (i.e., those with a lower surface-to-volume ratio) would be favored in drier environments since they have lower rates of evaporative water loss and, hence, are more resistant to desiccation^35,36^. In thermoregulating anurans, “the heat balance hypothesis” suggests that the higher heat retention capacity associated with a lower surface-to-volume ratio would benefit organisms with large body sizes in cold environments^31^. This hypothesis, analogous to the classic heat conservation explanation of Bergmann^37^ for size gradients in endotherms, can be extended to ectotherms below a certain body size threshold especially in the presence of compensatory physiological, morphological and/or behavioral mechanisms to reduce heating times^32,38^. For example, heating rates are faster in dark ectotherms than in light-colored ones, which is particularly advantageous for the survival of larger species under low ambient temperature conditions^39^. Finally, the habitat availability hypothesis associates the topography-macroclimate interaction, where body size variation may be influenced by habitat availability, where reduced areas (high mountains) may present decreases in body size in contrast to lowlands^40,41^.

In the context of body size evolution, it has been shown that there is no generalized pattern at the geographic and ecological level among anuran families^42^. This indicates that the determinants of changes in optimal body size are more related to clade-specific changes at a particular geographic scale for each anuran group. Thus, using an ecological and evolutionary approach, we evaluated the patterns of interspecific variation in body size using the amphibian genus *Pristimantis* as model study. *Pristimantis* is the most diverse vertebrate genus on the planet, with 584 known species^43^. Therefore, this genus is an excellent group for studying large-scale interspecific variations associated with body size due to its wide latitudinal and altitudinal distribution, from Central America to the Andean and Amazonian areas^44^, and from sea level up to 4500 m^45^. *Pristimantis* originated in South America during the early Miocene mainly associated with Andean orogenic events^46^. Studies on this genus have mainly been focused on taxonomic, systematic, and biogeographical contexts. However, the patterns related to interspecific variation in body size have only been partially evaluated^47^, with the existing information on body size being found across species descriptions, natural history notes, and selected morphological studies.

We used two approaches to investigate body size patterns. First, we collected body size data for all known *Pristimantis* species (584 species) without phylogenetic information. Second, we included phylogenetic information on 257 species for which body size data and molecular information were available for each species. We analyzed: (a) whether the SSD would conform to Rensch’s rule, where SSD should increase with body size when SSD is male-biased; (b) whether male and female body size and SSD are driven by environmental factors, about which we predicted that bioclimatic patterns would be positively associated with variation in body size; and (c) how the evolutionary rates of body size changed throughout evolutionary time with different patterns between males and females, for which we predicted a pattern of deceleration in the body size evolution rate alongside a decrease in both male and female body size.

## Results

### General pattern of SSD

**Figure 1:**
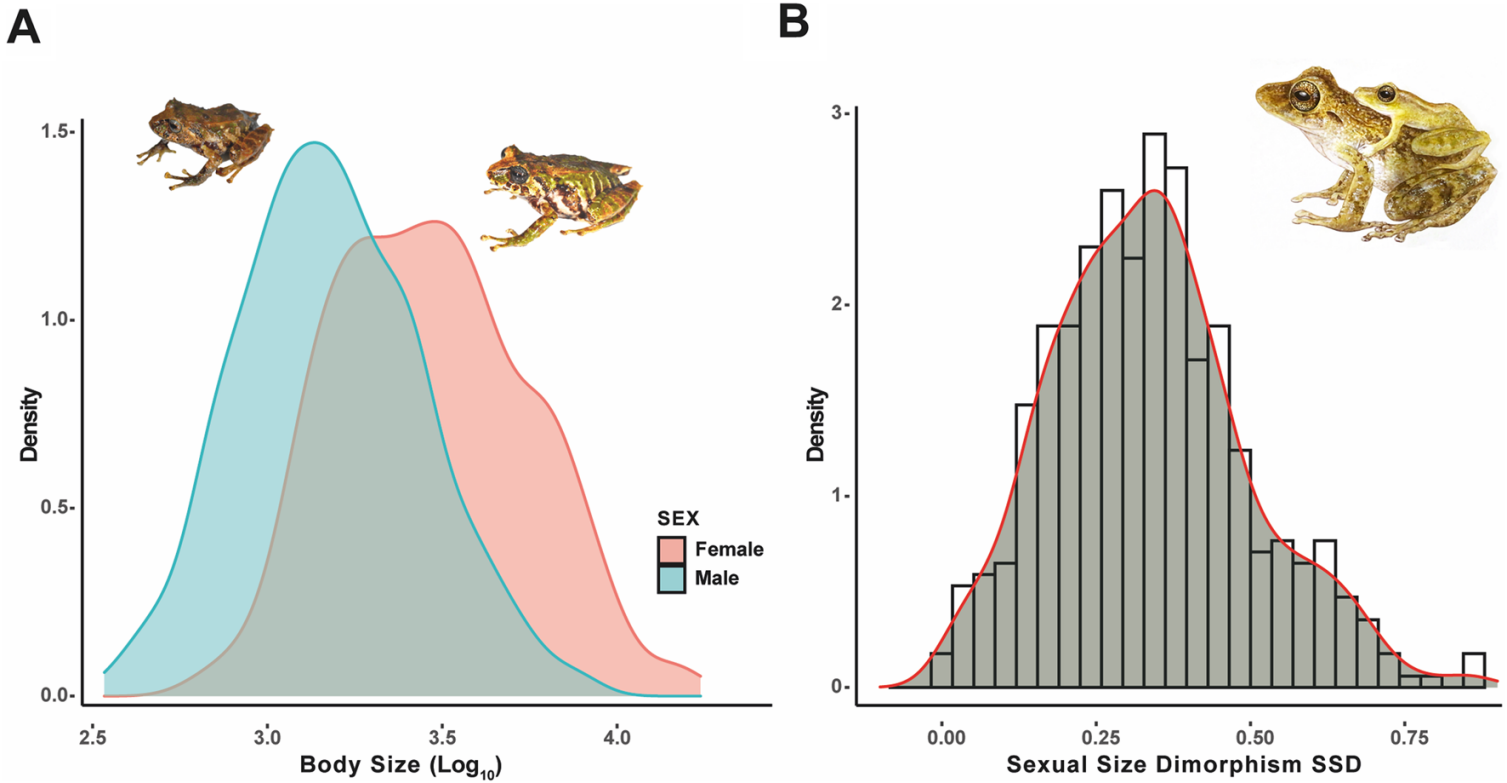
(**A**) Frequency distribution of the log10 maximum body size for males and females of rain frogs, genus *Pristimantis*. (**B**) Sexual size dimorphism with a fit of normal distribution curve. Images of *Pristimantis scoloblepharus* (Photos: Rivera, M.).

**Figure 2:**
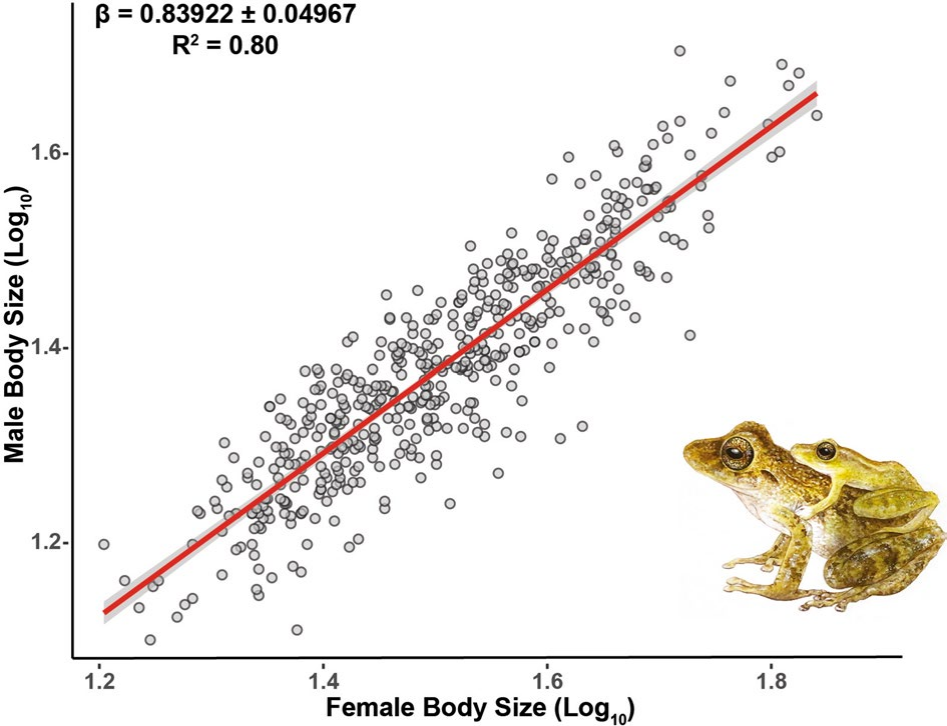
Linear regression with 95% confidence interval between the body sizes of males and females for 468 *Pristimantis* species.

Maximum body size varied considerably among *Pristimantis*, from 12.6 mm (*P. xeniolum*) to 50.8 mm (*P. labiosus*) in males and 15.3 mm (*P. coronatus*) to 69.3 mm (*P. lymani*) in females (Supplementary Table S1). Our results showed that 468 *Pristimantis* species evaluated in this study exhibited marked sexual dimorphism related to body size (Fig. 1A), while 27 species are monomorphic in body size (SDI < 0.1) (Supplementary Table S1). In turn, none of the species analyzed have a male-biased SSD. The species with the lowest SSD was *P. jabonensis* (0.003) and the highest was *P. latidiscus* (1.06) (Supplementary Table S1). On average, SDD was female-biased with a near-normal distribution (Fig. 1B). The linear regression between the body sizes of males and females showed that the allometric slopes were less than 1 (β = 0.83922 ± 0.04967, 95% CI 0.8027025–0.8757443; *R*^*2*^ = 0.80, *F*_*1,493*_ = 2038, *P* < 0.001; Fig. 2); this variation in SSD did not follow Rensch’s rule. Phylogenetically corrected analyses also showed a positive correlation between male and female sizes (*R*^*2*^ = 0.72, *F*_*1,255*_ = 686.4, *P* < 0.0001).

### Environmental predictors of body size

**Figure 3:**
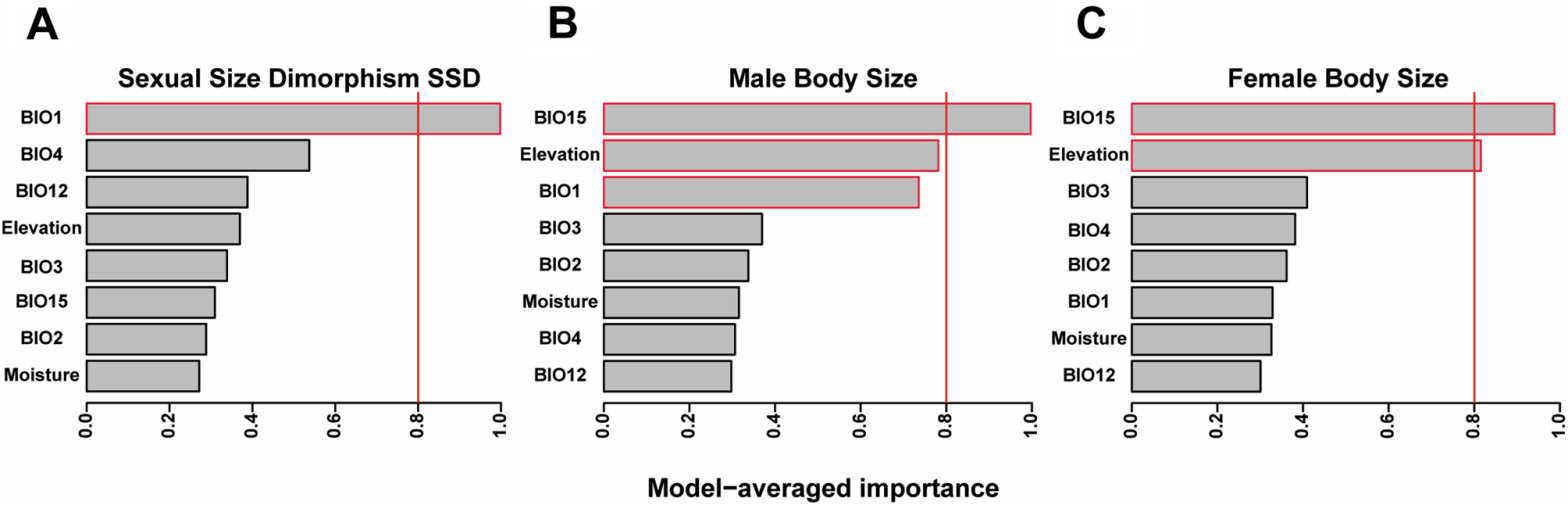
Relative importance of environmental variables according to the selection of models based on information theory. (**A**) Variables selected for SSD. (**B**) Variables selected for males. (**C**) Variables selected for females of rain frogs, genus *Pristimantis*.

Our analyses (with and without phylogeny) found significant patterns that showed that SSD and body size of males and females were associated with climatic variation related to the heat balance, water availability and habitat availability hypothesis. SSD analysis conducted by selecting models based on information theory identified temperature-associated variables (BIO1: annual mean temperature) as more relevant to explain SSD patterns in *Pristimantis*, with a greater relative importance of 0.99 supporting heat balance hypothesis (Fig. 3A; Table 1; Supplementary Table S6). For male body size, the best model included variables associated with precipitation (BIO15: precipitation seasonality, water availability hypothesis), elevation (habitat availability hypothesis), and temperature (BIO1, heat balance hypothesis), with relative importance values of 0.99, 0.78, and 0.73, respectively (Fig. 3B; Table 1; Supplementary Table S6). For females, model selection yielded a similar result (BIO15 and elevation). However, the relative importance values gave more weight to elevation, with 0.81 compared to males (Fig. 3C; Table 1; Supplementary Table S6). Based on the stepwise phylogenetic regression, the candidate model that best fitted the body sizes of males and females incorporated the variables BIO1, BIO2, BIO4 (heat balance hypothesis) and altitude (habitat availability hypothesis); for SSD, the best model included the variables BIO3 (heat balance hypothesis) and BIO15 (water availability hypothesis) (Table 2).

**Table 1:** Selecting models based on information theory indicating the combination of environmental variables for males, females, and SSD of rain frogs, genus *Pristimantis*.

Multivariate models	β	Std. error	95% CI (lower, upper)	Importance	AICc	w_i_
**SDI**						
BIO1	0.0434		(0.0212, 0.0656)	0.9980	− 379.7360	0.06398504
BIO4	− 0.0079		(− 0.0277, − 0.0119)	0.5372		
**SVL males**						
BIO15	0.0199	0.0053	(0.0096, 0.0302)	0.9970	− 770.9435	0.08821570
DEM	− 0.0156	0.0118	(− 0.0386, 0.0075)	0.7814		
BIO1	− 0.0138	0.0116	(− 0.0365, 0.0089)	0.7360		
**SVL females**						
BIO15	0.0180	0.0057	(0.0068, 0.0292)	0.9868	− 702.0437	0.05700275
DEM	− 0.0132	0.0089	(− 0.0306, 0.0043)	0.8154		
BIO4	0.0030	0.0053	(− 0.0074, 0.0135)	0.4093

**Table 2:** Best model by stepwise phylogenetic regression for males, females and SSD of rain frogs, genus *Pristimantis* indicated by the AICc value.

GLM models + phylogenetic tree	β	AICc
**SDI**		
BIO3	0.02712334	− 174.21
BIO15	0.01941823	
**Males**		
Bio1	− 0.02222096	− 503.39
Bio2	0.01660305	
Bio4	− 0.01595270	
DEM	− 0.02089806	
**Females**		
Bio1	− 0.01591907	− 479.2
Bio2	0.01979775	
Bio4	− 0.02465312	
DEM	− 0.01612496	

### The evolution of body size in *Pristimantis*

#### Ancestral state reconstruction

**Figure 4:**
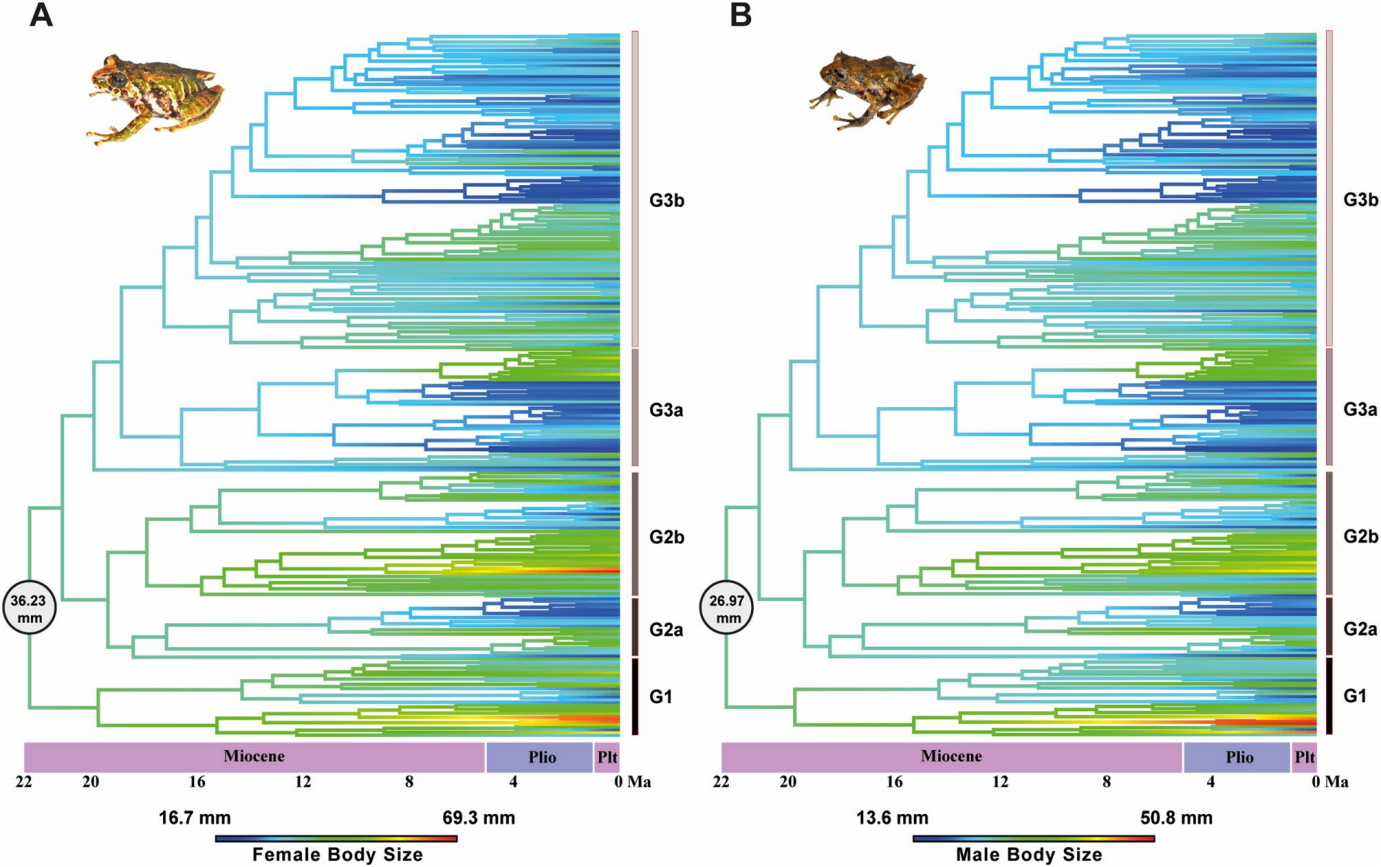
Maximum likelihood continuous-character ancestral reconstruction for the maximum body size for males (**A**) and females (**B**). The horizontal bar indicates the corresponding clade. The color gradient indicates the body size range of rain frogs, genus *Pristimantis*. Images of *Pristimantis scoloblepharus* (Photos: Rivera, M.).

There were significant phylogenetic signals for the body size of males (λ = 0.85, *P* < 0.001) and females (λ = 0.86, *P* < 0.001). Maximum likelihood continuous-character ancestral reconstruction for the maximum body size yielded an internal node value of 36.23 mm for females (95% CI 28.36–44.10) (Fig. 4A), while for males it was 26.97 (95% CI 21.42–32.51) (Fig. 4B). The general pattern showed that most of the body sizes among the different clades were close to the average size (female = 33.78, male = 25.19, SSD = 0.34) (Table 3).

**Table 3:** The major phylogenetic clades associated with body size ancestral states, body size evolution rates, age of nodes and ecoregions for males, females, and SSD of rain frogs, genus *Pristimantis*.

Clade	Body size ancestral states			Body size evolution rates		Age of the Major clade (Ma)	Ecoregion
	Male	Female	SSD	Male	Female		
G1	28.53, 95% CI 22.74–34.33	38.46, 95% CI 30.24–46.69	0.35, 95% CI 0.16–0.54	5.6506	9.8752	19.872	Northwestern Andes, Chocó, Central America, Amazonia, Colombian Eastern Andes
G2a	26.14, 95% CI 21.06–31.23	35.38, 95% CI 28.17–42.59	0.35, 95% CI 0.19–0.52	5.2750	9.0240	18.532	Central Andes, Northwestern Andes, Venezuelan Andes, Chocó, Amazonia
G2b	26.75, 95% CI 22.08–31.43	36.73, 95% CI 30.10–43.37	0.37, 95% CI 0.22–0.53	5.4816	11.1643	18.014	Chocó, Amazonia, Colombian Eastern Andes, Guiana Shield, Central Andes, Atlantic Forest, Northern Sudamerican montane coastal
G3a	23.70, 95% CI 18.67–28.72	31.57, 95% CI 24.44–38.70	0.33, 95% CI 0.17–0.49	3.2704	6.9802	16.684	Northwestern Andes, Colombian Eastern Andes
G3b	24.74, 95% CI 20.55–28.92	32.71, 95% CI 26.76–38.65	0.32, 95% CI 0.19–0.46	3.0947	6.4955	17.355	Northwestern Andes, Amazonia, Central Andes, Central America, Guiana Shield, Colombian Eastern Andes, Chocó, Venezuelan Andes

#### Evolutionary rate of body size

**Figure 5:**
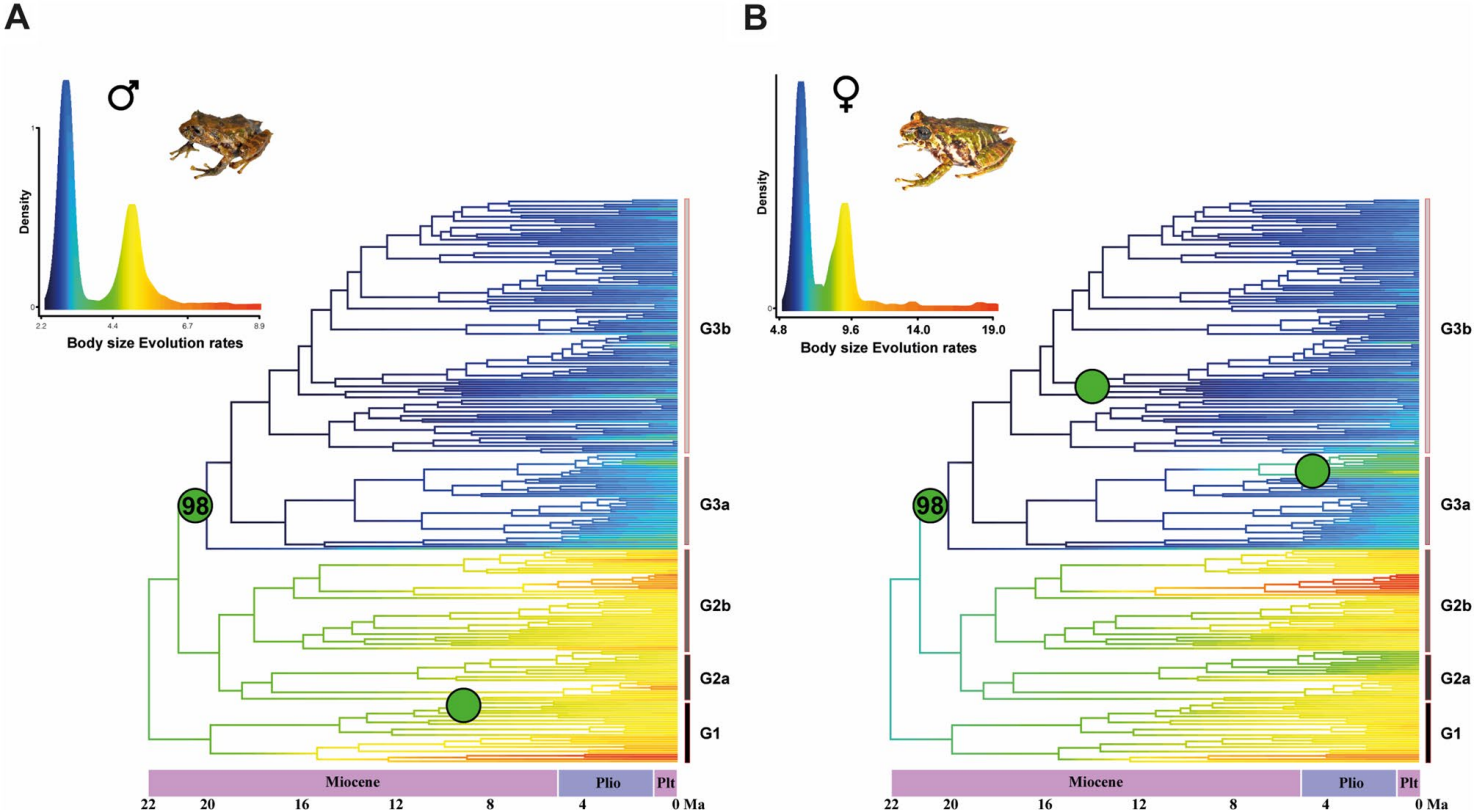
Bayesian analysis of macroevolutionary mixtures (BAMM) for males and females showing changes in body size evolution rates (green dots) for males (**A**) and females (**B**) of rain frogs, genus *Pristimantis*. Top of each phylogenetic tree with the histogram represented by the density of the body size evolution rates. The horizontal bar indicates the corresponding clade. Images of *Pristimantis scoloblepharus* (Photos: Rivera, M.).

**Figure 6:**
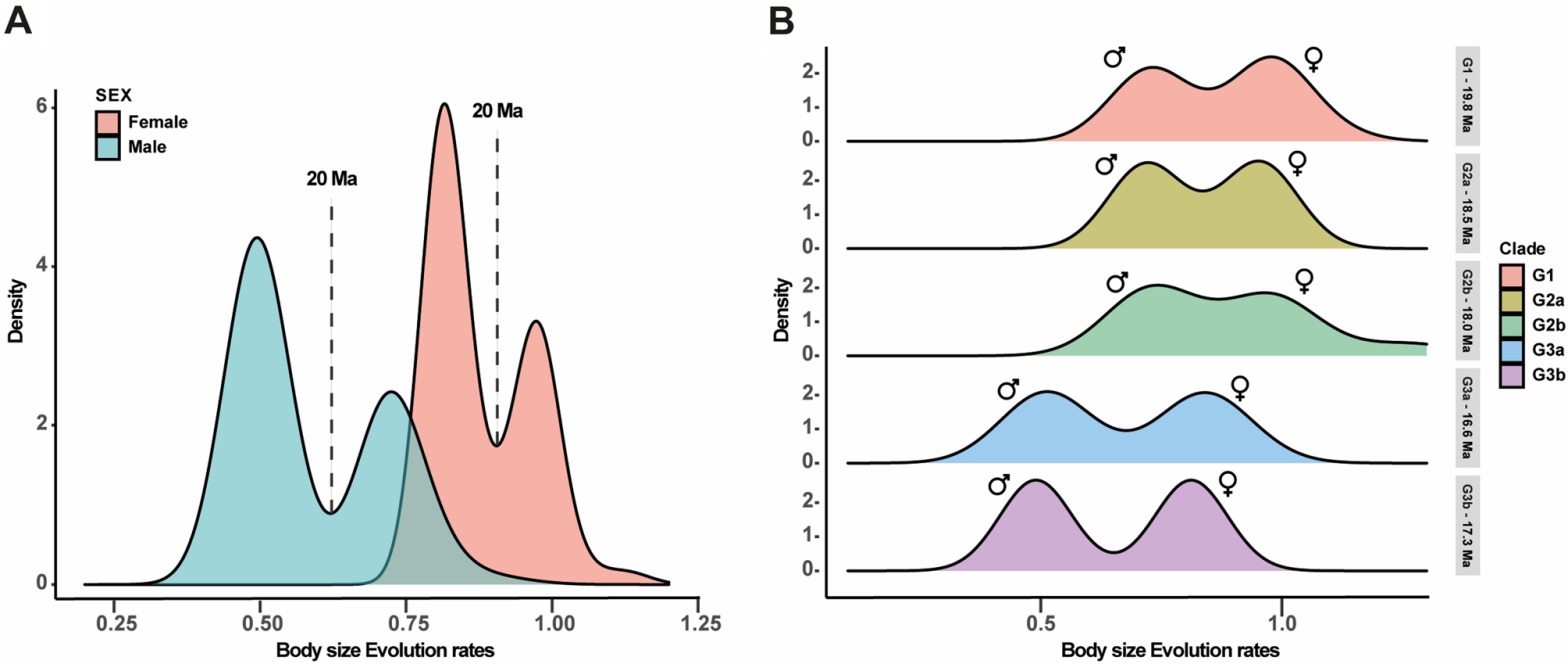
(**A**) Histogram showing the density of body size evolution rates for males and females of rain frogs, genus *Pristimantis*. Dotted line indicates the age (Ma) that represents the change in the rate regime (Fig. 5). (**B**) Histograms by phylogenetic clades showing the density of body size evolution rates for males and females, the horizontal bars indicate the clade with its age (Ma).

Bayesian analysis of macroevolutionary mixtures (BAMM) supported changes in the evolution of body size, with the rates for males and females having a marginal probability of 0.988 (> 95% of all models sampled from the post-burn-in chain). A regimen change located at node 98 (20 Mya) between clades G1, G2, and G3 occurred in males and females. Regime change was associated with a reversal in the rate of body size evolution; thus, within these clades, the rates tended to decelerate over time (Fig. 5A,B; Supplementary Table S7). In turn, males and females showed different patterns in the rates of body size evolution, where females had higher evolutionary rates than males, and for both sexes there was evidence of decelerating rate of body size evolution (Fig. 6A,B; Supplementary Table S7).

## Discussion

*Pristimantis* body size patterns exhibited marked SSD. However, the genus conforms to a female-biased inverse Rensch’s rule (b < 1). This is consistent with previously documented patterns for the three amphibian orders^47,48^. This deviation of SSD towards females could be related to fecundity selection, which describes the fitness advantages that result from the selection of traits that increase the number of offspring per reproductive period^48–50^. However, despite knowing that all *Pristimantis* species have direct egg development, the clutch size of most species is unknown, although estimates for a few species have been between 30 and 38 eggs per clutch^51–53^. Other explanations have attributed the variations in body size between males and females to the energy requirements associated with reproductive behavior. In males, investment is usually related to territorial defense, agonistic, and advertising behavior, whereas females invest more in body growth^49^. Accordingly, variations in body size have several ecological and evolutionary implications. For example, larger amphibians tend to have lower metabolic rates for maintaining their temperature compared to smaller amphibians^54^.

However, our results suggest that environmental conditions related with temperature (heat balance hypothesis) are associated with body size differences between males and females. SSD in *Pristimantis* exhibits a positive relationship for temperature seasonality (BIO4), which is a measure of temperature change over the year^55^, and annual mean temperature (BIO1), which is the average maximum and minimum temperature for the warmest and coldest months, respectively^55^.

Previous studies have shown that temperature seasonality is related to body size in amphibians, and this relationship would be associated with the phenomenon that shapes latitudinal gradients^48,56^. However, over 60% of *Pristimantis* species are distributed in Andean areas, where local temperature variations tend to be relatively stable^57^. Therefore, the annual mean temperature may be more related to SSD patterns in *Pristimantis*, especially because this variable presented an importance of 0.99, which indicates that the variable has appeared with high weights repeatedly among the different candidate models. However, around 18% (89) of the species are restricted to the páramo zone (> 3000 m a.s.l.), which includes more fluctuating thermal landscapes that can influence the physiological patterns relevant to body size differentiation^58^ that varies between 12 and 50 mm. Our data support a slight negative relationship between annual mean temperature (Bio1) and SSD (*R*^*2*^ = 0.05, F_*4,84*_ = 2.304, *P* = 0.009) for species above 3000 m a.s.l.

The climatic predictors that best explain body size for each sex are precipitation seasonality (water availability hypothesis) and elevation (habitat availability hypothesis). Although mean annual precipitation is used most often to describe different ecological relationships, the dynamic of temporal variation in precipitation under different geographical settings can influence ecosystem responses and the morphological trait patterns of species^59^. The elevation is also associated with body size in *Pristimantis*, especially in females, possibly due to the habitat availability hypothesis, which predicts larger body sizes in lowlands relative to mountains^41^. In turn, body size has been associated with habitat variability^60^ and as a predictor of brood size^61^. For example, in high-altitude areas, it has been shown that breeding seasons are shorter for some amphibian species. Consequently, the number of clutches is lower^62^, which could result in smaller body sizes. However, these aspects remain to be explored in the case of *Pristimantis*, for which data associated with the number of clutches are still incipient. Therefore, possible relationships between clutch size, altitude, body size, and Andean bioclimatic landscapes remain as hypotheses to be evaluated.

### Evolution of *Pristimantis* body size

**Figure 7:**
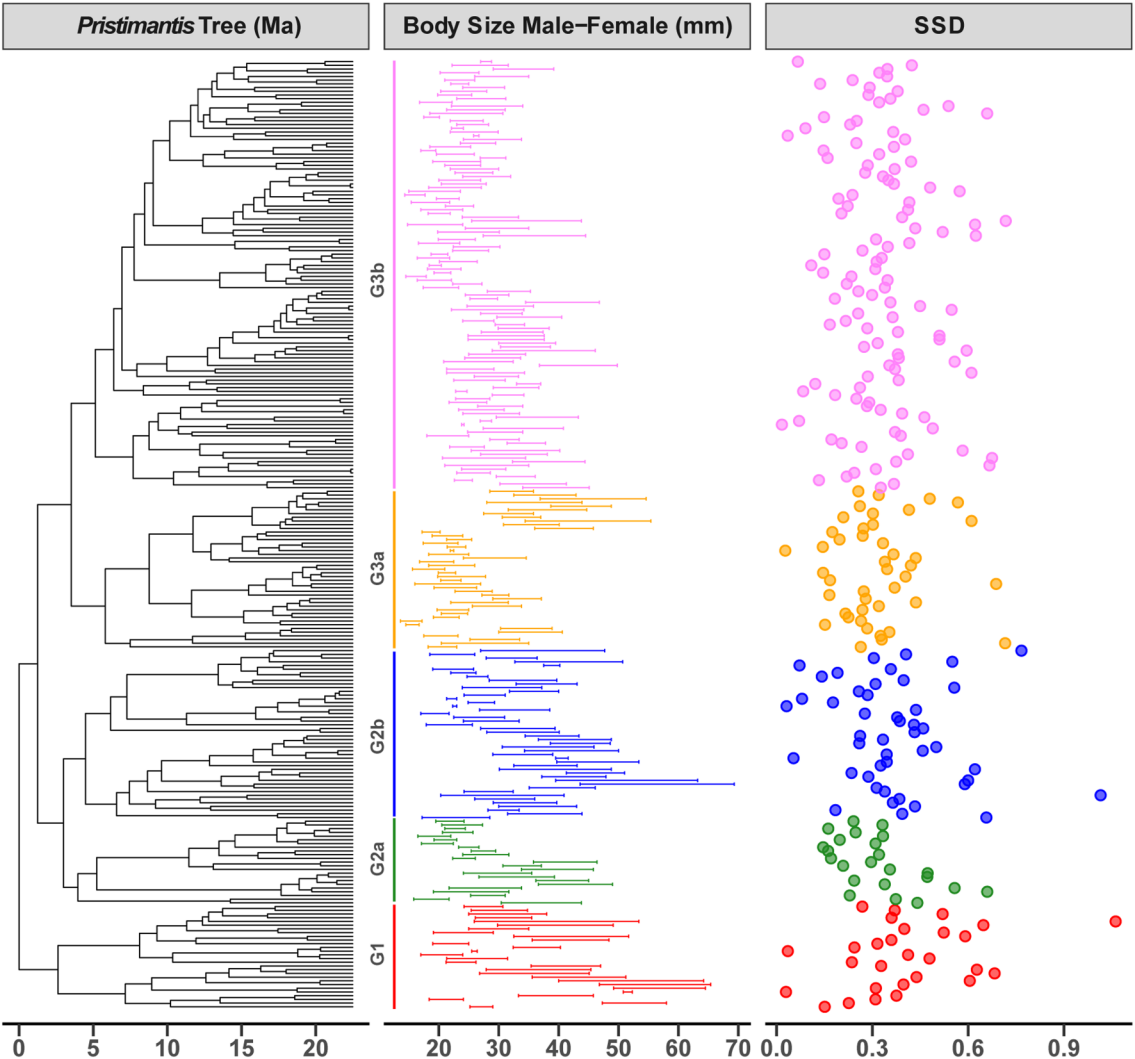
Calibrated phylogeny of rain frogs, genus *Pristimantis* with the range of body size for males and females and SSD. Each color represents a clade.

Body size of *Pristimantis* species ranged from 12.6 to 50.8 mm for males and between 15.3 and 73 mm for females. A recent study by^47^ reported *P. nanus* (not included in this study) as the smallest species, with males being only 12.4 mm. This new species is part of the *P. trachyblepharis* species group, which is a group of micro-endemic taxa distributed in south-central Ecuador and northern Peru^47^. Our analyses did not identify any clades with a tendency to miniaturize, as size patterns varied within each clade (Fig. 7; Supplementary Fig. S2). This implies that the species within each clade present multiple selective pressures that have determined the wide ranges of body size in *Pristimantis*. The tendency toward miniaturization or gigantism in anurans is more related to clade-specific patterns associated with responses to ecological and geographic factors^42^. However, among clades with different reproductive modes, miniaturization has been shown to be the main pattern in direct-developing amphibians (the only mode in *Pristimantis*)^42^.

The phylogenetic signal of body size for males and females was high (Pagel’s λ > 0.85), which supports the findings previously reported by Zumel^47^ and those reported for amphibians in general^63^. However, for other neotropical amphibians, such as the genus *Boana* which has a similar distribution to *Pristimantis*, a low phylogenetic signal for species has been reported, having large variations in body size with random patterns of trait variation^33^. Nevertheless, the strong phylogenetic signal found for *Pristimantis* sexes indicates that the data fit a Brownian motion model well, indicating that closely related species tend to have similar body size^64,65^.

### Evolutionary rate of body size

Although our phylogeny is incomplete, body size evolution rates for male and female *Pristimantis* suggested a regimen change associated with progressive rate deceleration, with a break between clades G1–G2 and G3 (Fig. 5A,B; Table 3). It is important to clarify that for estimation of diversification rates (speciation-extinction analyses), incomplete sampling of taxa may have an impact on rate estimates^66^. However, for modeling trait evolution, it has no implications if taxon sampling is random with respect to phenotypes^67^. Changes in body size evolution rates of *Pristimantis* may be associated with historical biogeographic patterns that have shaped its diversification patterns. Clades G1–G2 showed the highest ranges of body size evolution rates, between 5.2750 and 5.6506 for males and 9.0240–11.1643 for females (Table 3). These early clades consist mainly of species from the ecoregions of the Northwestern Andes ecoregions, which have been suggested as a possible center of origin of *Pristimantis*^68^. Subsequent dispersal patterns with the colonization of ecoregions such as Chocó, Amazonia, the Colombian Eastern Andes, Central Andes, the Venezuelan Andes, the Atlantic Forest, Northern South American montane coastal areas, and the Guiana Shield have shaped clades G1–G2 (Acevedo et al., unpublished). Clade G3 (Fig. 5A,B; Table 3) exhibited evolution rates with a tendency to slow down between 3.2704 and 3.0947 Ma for males and 6.9802–6.4955 Ma for females (Table 3). Clade G3 comprises species from the Northwestern Andes, the Colombian Eastern Andes, Amazonia, Central Andes, Central America, and the Guiana Shield. The deceleration pattern is explained by the trend towards an evolutionary optimum in body size that *Pristimantis* species have experienced, where forces acted to reduce phenotypic variation. In contrast, the initial acceleration may be the response of the ancestral lineages to some selective pressure experienced in the early stages of genus diversification.

Females had higher evolutionary rates than males (opposite of Rensch’s rule). For both sexes, there was a pattern of deceleration in the rates of body size evolution, and this was associated with reduced body size (Fig. 6A,B). This finding is in contrast with patterns found in mammals, for example, where the deceleration of body size evolution rates is associated with an increase in size^69^. However, at the macroevolutionary level for different amphibian groups, the patterns of the evolutionary rates of traits associated with body size are not yet clear. Therefore, future studies must test ecophysiological hypotheses, niches, and other ecological attributes to obtain a general understanding of the changes in the patterns of body size evolution rates. Our results provide an approach for understanding the relationship between body size and its evolutionary rates in *Pristimantis*.

In conclusion, our results support the evolution of body size and SSD variation in *Pristimantis*, suggesting that environmental variables can drive and/or maintain the degree of body size divergence between sexes. The rates of body size evolution show a deceleration over time with a slight increase in body size. These results contribute to knowing the factors involved in body size and its evolution, helping to generate future hypotheses related to sexual and natural selection or macroevolution tendency in SSD.

## Methods

### Datasets

We collected data on maximum body size, represented by snout-vent length (SVL), for adult males and females of all known *Pristimantis* species (584) described until July 2021. Maximum size was taken as a proxy for the potential size of organisms with indeterminate growth^54,70,71^, a common standard in studies that explore interspecific size comparisons in anurans^31,32,42^. Body sizes were log-transformed (log_10_) to reduce the asymmetry of the data since they cover a wide range of body size values. The species included in this study represented the entire known distributional range for the genus, spanning from Central America to the Central Andes of Peru and Bolivia. Measurement data were compiled from the original descriptions of each species, field data, and museum records on the review and verification of specimens (Colección Herpetológica del Instituto de Ciencias Naturales, Universidad Nacional de Colombia; Colección Herpetológica de la Universidad de Pamplona; Museo de Historia Natural de la Universidad Distrital Francisco José de Caldas; and the Museo de Historia Natural de la Universidad de los Andes). Measurements were taken using a digital caliper to the nearest 0.01 mm following^72^. The limited information in the literature regarding body size for multiple males or females per *Pristimantis* species did not allow us to perform additional analyses to determine the influence of intraspecific sampling and its implications on SSD estimates.

We built two databases for developing the two analytical approaches. The first approach focused on ecological analysis without including a molecular phylogeny, and the final database included 495 species (85% of the known species of the genus) the remaining 89 species only present data for one of the sexes, therefore they were excluded (Supplementary Table S1). For the second database, we used a subset of species on which we performed additional evolutionary analyses. Although there are molecular data available in different public repositories (e.g., Genbank) for approximately 300 *Pristimantis* species, we only included 257 species for which there were molecular data and body size measurements were available for both sexes (Supplementary Table S2).

To estimate the SSD for each species and to evaluate Rensch’s rule, we used the size dimorphism indices (SDI's) proposed by Lovich and Gibbons^73^:

$$\begin{aligned} {\text{SDI}} & = \left( {{\text{size}}\;{\text{of}}\;{\text{largest}}\;{\text{sex/size}}\;{\text{of}}\;{\text{smallest}}\;{\text{sex}}} \right) + 1\;{\text{if}}\;{\text{males}}\;{\text{are}}\;{\text{larger}} \\ {\text{SDI}} & = \left( {{\text{size}}\;{\text{of}}\;{\text{largest}}\;{\text{sex/size}}\;{\text{of}}\;{\text{smallest}}\;{\text{sex}}} \right){-}1\;{\text{if}}\;{\text{females}}\;{\text{are}}\;{\text{larger}} \\ \end{aligned}$$

### Divergence time estimation

The divergence times for the 257 species (Supplementary Table S3) that were included in the body size matrix were obtained from a concatenated data set for 304 *Pristimantis* species for six partial mitochondrial genes (12S, 16S, CYTB, COI, ND1, and ND2) and two partial nuclear genes (RAG1 and TYR). Two hundred sixty-one species were retrieved from GenBank (as of August 2020). The remaining sequences comprised 43 previously unsequenced *Pristimantis* species (unpublished data), of these 24 were included in the final body size matrix.

We selected the best partition scheme and the corresponding substitution models using PartitionFinder 2^74^ with the Bayesian information criterion and the greedy algorithm^74^. We assumed 19 possible partitions through the concatenated data matrix (by genes and codons).

Divergence times were estimated using a relaxed Bayesian clock implemented in BEAST 1.10.4^75^, using a Yule speciation process^76^ with substitution rate variation. The analysis was performed using 100 million generations that were sampled every 1,000 generations. We based our analysis on four previously published divergence times: (a) the divergence time between eleutherodactylines and the South American clades of *Pristimantis*, 36.52 Mya (I.C. = 26.56–50.81)^45^; (b) the most recent common ancestor of *Pristimantis*, 24.45 Mya (I.C. = 17.30–34.82)^45^; (c) the divergence age of the species of *P. pardalis*, 8.6 Mya (I.C. = 5.5–12.0)^46^; and (d) the clade age of *P. taeniatus*, 8.3 Mya (I.C. = 5.6–11.2)^68^. The phylogeny was rooted including four outgroups: *Tachiramantis*, *Oreobates*, *Eleutherodactylus*, and *Craugastor*^45^. The trees were visualized using Figtree v.1.3.1^77^. Subsequently, the calibrated tree was pruned using the 'drop.tip' function of the 'ape' package by removing the species not included in the final body size matrix (Supplementary Tables S2, S3).

### Cross-species analyses

We used a cross-species approach in which each species represented independent data^78^. We created an extensive compilation of the latitudinal and longitudinal coordinates of the 495 species. The geographical occurrence data were obtained through different methods, such as from the Global Biodiversity Information Facility (https://www.gbif.org), the scientific literature, a review of biological collections (as done for body size), and our own field data. Records for each species were mapped in QGIS v3.14.16^79^ and individually curated to correct georeferencing errors and eliminate erroneous locations. The final database contained 9237 geographic records (Supplementary Table S4 includes geographic information for all *Pristimantis* species).

We selected 24 climate variables at a resolution of 1 km^2^, taken from Worldclim (https://www.worldclim.org) and ENVIREM (https://envirem.github.io). We excluded highly correlated variables (Pearson correlation coefficient ≥ 0.60) from the analyses to minimize multicollinearity using Spearman’s rank correlation performed in the corrplot R package^80^ (Supplementary Fig. S1; Table S5). The resulting variables were used to test the two main hypotheses: (a) heat balance^31,81^: annual mean temperature (BIO1), mean diurnal range (BIO2), isothermality (BIO3), and temperature seasonality (BIO4); and (b) water availability^36^: annual precipitation (BIO12), precipitation seasonality (BIO15), and moisture index. Additionally, we included altitudinal information to estimate the available habitat formed by the interaction between the topography and macroclimate^41,82^. For each species, the variables were averaged based on the geographic occurrences (Supplementary Table S1). The predictors were logarithmically transformed and then scaled to have a mean of zero and unit variance using the 'standardize' package in R^83^.

### Phylogenetic comparative analyses

We estimated the phylogenetic signal of the residual errors for the body sizes of males and females using the statistic lambda λ (Pagel’s λ). When λ = 1, it signifies that trait relationships are equal to the correlation of species imposed by their shared evolutionary history^84^. When λ = 0, it signifies patterns of trait similarity between species that are independent of phylogeny (non-phylogenetic regression)^85,86^. The phylogenetic signal tests were implemented in R using the ‘phylosig’ function of the ‘phytools’ package^87^.

We mapped the ancestral states of male and female body size that were transformed to the log10 scale for the internal nodes of the calibrated phylogeny obtained in BEAST. We used the ‘contMap’ function in the R ‘phytools’ package^87^. This function uses the maximum probability to estimate the ancestral states, with the trait data for each species at the tips of the tree^88^.

### Bayesian analysis of macroevolutionary mixtures

We performed an analysis in BAMM v. 2.5.0^89^ to estimate and quantify the heterogeneity in the evolutionary rates of body size in males and females (rate of phenotypic evolution, *ß*). BAMM uses reversible-jump Markov chain Monte Carlo (rjMCMC) to choose between models that vary in the number of evolutionary regimes^90,91^. The priors for males (poissonRatePrior = 1.0, betaInitPrior = 0.054371, betaShiftPrior = 0.051182) and females (poissonRatePrior = 1.0, betaInitPrior = 0.027243, betaShiftPrior = 0.051182) were estimated using the BAMMtools v. 2.1.7 package^89^ in R before analysis in BAMM. We used a consensus tree obtained previously in BEAST, and the chains were run for 100 million generations with a sampling frequency of 10,000. The first 10% of samples were discarded as burn-in. An effective sample size (ESS) > 200 of the log-likelihood and the number of shift events present in each sample were evaluated using the R package ‘coda’^92^. The 95% credible set of distinct rate-shift configurations (CSSs) was obtained using the BAMMtools v. 2.1.7 package^89^ and visualized on the calibrated tree.

### Ecological and evolutionary analysis

To evaluate whether SSD patterns related to the increase, decrease, or isometry of the maximum log10-transformed *Pristimantis* body size, we performed simple linear regressions using the ‘lm’ function in R^93^ for the database that included all species. We also performed a phylogenetic generalized least squares (PGLS) regression including the calibrated phylogenetic tree. For both analyses, we placed females on the x-axis (dependent variable)^15^. We examined the slope of the regressions of male and female body size according to three scenarios following Fairbairn^94,95^: (a) Rensch’s rule, slope greater than one (b > 1); (b) inverse of Rensch’s rule, slope less than one (b < 1); and (c) isometry, slope equal to one (b = 1). Additionally, we performed a simple linear regression comparing the SSD values against the SVL log10 of males and females to evaluate the increase or decrease in SSD according to sex.

To determine the predictors that best explained the interspecific variation in body size, we performed two analyses, one with the database for the 495 *Pristimantis* species (Supplementary Table S1) and another one including phylogenetic information and male and female body size of 257 species (Supplementary Table S2). Analyses were performed separately for males, females, and SSD. For the first analysis (495 species), we selected models based on information theory criteria^96^. We used the R ‘glmulti’ package^97^, which finds the set of confidence models among all possible models. The best models were found by a genetic algorithm (GA), from which the multi-model average was derived using the ‘coef’ function. The GA incorporates an immigration operator, which allows the eliminated variables to be reconsidered. Immigration increases the level of randomization and, therefore, the probability of convergence of the model to a global optimum rather than a local one^96,97^. We obtained the importance of the evaluated predictors, which was equal to the sum of the weights / probabilities of the models in which the variable appeared. Then, a variable with large weights that appeared in a set of candidate models received a high importance value^98^. We set the parameter level to 1 to include only the main effects, meaning that 28 represented the 256 possible models that were generated in the set of considered candidates. We used the function ‘crit = aicc’ (AICc or AIC corrected) to select the model and the multi-model inference, which were based on different predictors, accounted for all possible models that corresponded to different biological hypotheses, and varied in the degree to which they fit the available data^99^.

The second analysis included a stepwise phylogenetic regression using the calibrated phylogeny and the databases for the 257 species that included the body size of males, females, and SSD in conjunction with the previously defined climatic variables (Supplementary Table S2). The stepwise search mode was backward elimination starting from the full model, including all independent variables (BIO1 + BIO2 + BIO3 + BIO4 + BIO12 + BIO15 + DEM). The models were compared based on their AIC values.

### Ethics statement

All procedures related to sampling, processing, and obtaining genetic material of some amphibian species included in this study were evaluated and approved by the Research Ethics and Safety Unit of the Pontificia Universidad Católica de Chile (protocol ID 180611004). Collection permits were approved under the scientific research framework agreement No: 200 of the Corporacion Autonoma Regional de la Frontera. The methods developed in this manuscript do not involve live animal experimentation.

## Supplementary Information

Supplementary Figures.

Supplementary Table S1.

Supplementary Table S2.

Supplementary Table S3.

Supplementary Table S4.

Supplementary Table S5.

Supplementary Table S6.

Supplementary Table S7.

## Data Availability

The datasets generated and/or analysed during the current study are available in the Supporting information section. Supplementary Table S3 have the *Pristimantis* species included in the phylogenetic analyses with the respective Genbank accession numbers linked to the NCBI website.
