# Supplementary material for: Ecological and evolutionary trends of body size in Pristimantis frogs, the world's most diverse vertebrate genus
Source: Sci Rep. 2022 Oct 27;12:18106. doi: 10.1038/s41598-022-22181-5 (PMC9613995; doi:10.1038/s41598-022-22181-5)
Supplement: Supplementary file 1 — Supplementary Figures. [file 41598_2022_22181_MOESM1_ESM.pdf]

## SUPPLEMENTARY FIGURES

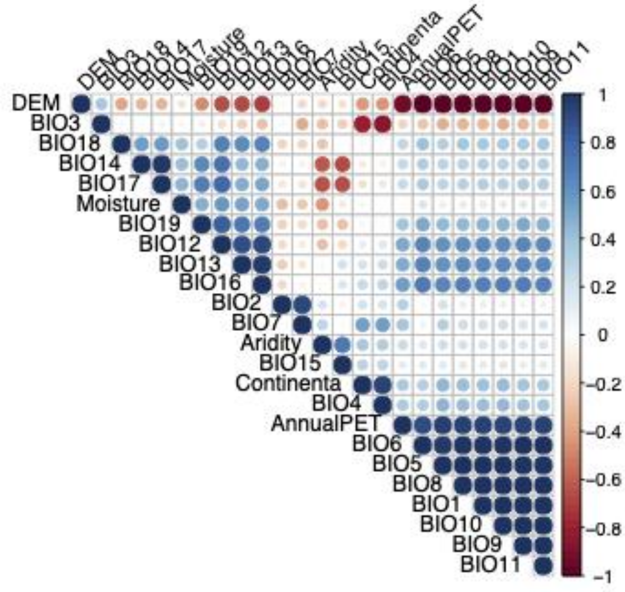


**Figure S1.** Pearson's correlation coefficients of the relationship between environmental variables.

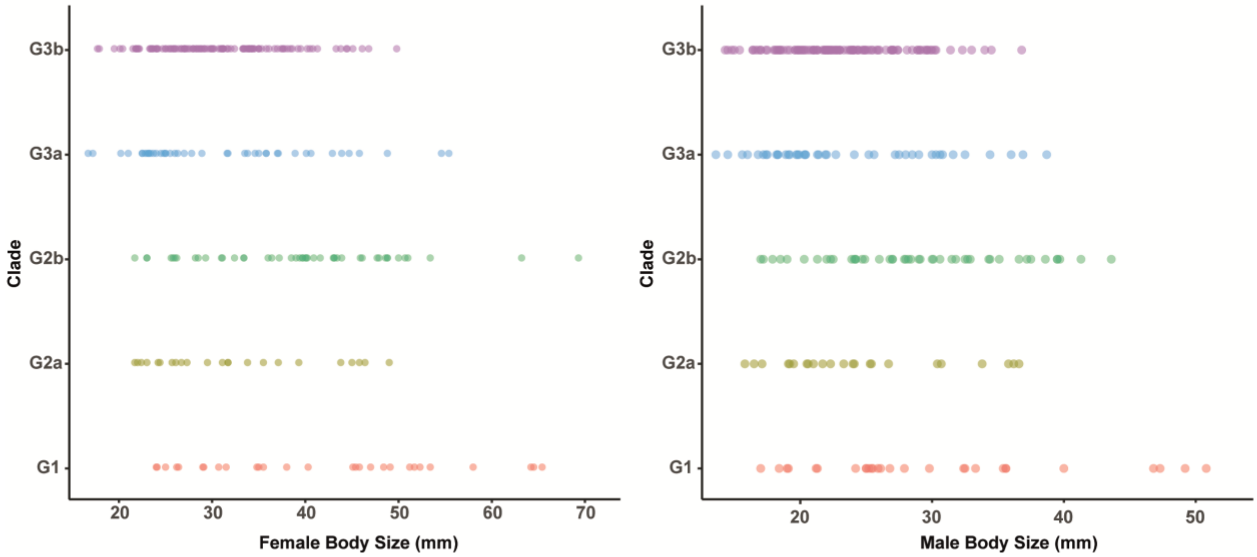


**Figure S2.** Body size ranges according to the major phylogenetic clades for females (A) and males (B)
